# Prognostic value of HPV 16/18 genotyping and geminin mRNA quantification in low-grade cervical squamous intraepithelial lesion

**DOI:** 10.1080/21655979.2021.2009959

**Published:** 2021-12-07

**Authors:** Ziwen Zheng, Xiaorong Yang, Xinyu Yao, Ling Li

**Affiliations:** a Department of Gynecologic Oncology, JiangXi University, JiangXi, China; b Department of Oncology, JiangXi University, Nanchang, China

**Keywords:** HPV16/18, LSIL, cervical intraepithelial neoplasia, loop electrosurgical excision procedure

## Abstract

Low-grade cervical squamous intraepithelial lesion is a precancerous neoplasia that has appreciable probability to evolve into malignancy. To explore the prognostic value of HPV 16/18 genotyping and geminin mRNA quantification in predicting the progressiveness of LSIL. We recruited 212 participants who were negative for intraepithelial lesion or malignancy (NILM 76), low-grade squamous intraepithelial lesion (LSIL 85), high-grade squamous intraepithelial lesion (HSIL 36) and cervical intraepithelial neoplasia grade cervical cancer grade 3, (CIN3 15) patients. Tissues were obtained during excisional treatment. HPV 16/18 genotyping and geminin mRNA qRT-PCR were performed. HPV 16/18 positivity rate and geminin mRNA level were integrated with the clinical parameters into a multivariate logistic model. Area under curve was yielded based on receiver operation curve derived from this multivariate logistic model. Follow-up visits were performed to LSIL patients with progression. HSIL patients have higher HPV 16/18 positivity rate and geminin mRNA levels than LSIL. Among HSIL, CIN3 have higher HPV 16/18 positivity rate and geminin mRNA levels. Multivariate logistic analysis showed that HPV 16/18 positivity and geminin mRNA expression status are independent factors for differentiating HSIL and LSIL. The baseline HPV 16/18 positivity rate and geminin mRNA levels of 18 LSIL patients who developed HSIL are significantly higher than non-progressive LSIL patients. The values examined at follow-up timepoints were also higher than baseline. These results suggest that geminin is implicated in the progression of LSIL and combining HPV 16/18 genotyping and geminin mRNA qRT-PCR could potentially differentiating the progressive LSIL and improve the efficacy of clinical intervention.

## Introduction

Cervical cancer is a common malignancy that originates in women’s reproduction system, which posts a threat on women’s life and well-being [1]. Recent statistics showed that there are 500,000 women affected by cervical carcinoma per year, and China has 130,000, accounting for 1/3 world-wide incidence [2]. Cervical carcinoma is usually preceded by long period of cervical intraepithelial neoplasia (CIN), also termed squamous intraepithelial lesion (SIL) [3]. The early diagnosis and treatment of SIL is of great importance for reducing the incidence of cervical carcinoma. Depending on the severity of histological abnormalities, SIL can be graded as low grade or high grade SIL [4]. Some SIL is heterogeneous and comprises of both lesions. Low-grade squamous intraepithelial lesion (LSIL) presents productive viral infection and corresponding clinical measures will be in place. Around 70–80% LSIL are able to regress spontaneously within 1–2 years, but 30% LSIL are refractory and 10% LSIL patients will develop high grade SIL (HSIL) that could evolve into a malignancy [5]. According to cervical carcinoma screening guideline of NCCN, clinical observations are recommended for treatment of LSIL, accompanied with close follow-up Pap tests and colposcopic examinations until the lesions resolve. Whereas the treatment of HSIL follow excisional treatment. Since risks persist when proper excision was lacking for progressive LSIL, some hospitals offer loop electrosurgical excision procedure (LEEP) to majority of LSIL patients to prevent the progression [6,7]. However, such decision would lead to over-treatment for transient LSIL patient that may cause occasional iatrogenic complication, and thus posting great economic pressure on patient, hospital and the society as a whole [8]. Therefore, differentiation of LSIL of high progressive potential is critical in subsequent intervention of LSIL.

Currently, cytology is the most reliable method to predict progression of LSIL. Normal cytology (negative for intraepithelial lesion or malignancy (NILM)) or mildly abnormal cytology, i.e. atypical squamous cells of undermined significance has relatively low 5-year progression rate, while more severe cytological abnormalities, i.e. atypical squamous cells or atypical glandular cells, holds substantially higher progressive risks. However, 10% progression was developed from LSIL with normal or mildly abnormal cytology [3]. In clinical practice, the inter-rater variability often confounds the ascertainment of progressive LSIL [9–11]. This dilemma necessitates the search for effective biomarker to predict the behavior of LSIL.

Human papilloma virus (HPV) is the most common sexually transmitted infection in the world, which is also the most prevalent cause of cervical carcinoma and CIN [12]. In recent decade, multiple researches have found that HPV DNA screening could reduce the incidence and mortality of cervical carcinoma. HPV 16/18 are two most common strains that cause human genital cancer. HPV 16/18 positivity has been reported to elevate the risk of progression irrespective of cytological findings [13]. American Society for Colposcopy and Cervical Pathology (ASCCP) management guidelines suggest that HPV 16/18 positivity is an independent indicator for colposcopy referral for women elder than 30 years of age [14]. From screening aspects, HPV 16/18 genotyping became the most sophisticated strategy. However, prospective study investigating HPV 16/18 positivity in LSIL progression. Geminin has been reported as a negative regulator during DNA replication, which maintains chromosomal integrity by blocking CDT1 [15]. Emerging evidence implicated that geminin playsa dual role in tumorigenesis and cancer development. Dysfunction of geminin could promote DNA replication and apoptosis in cancer cells [16]. On the other hand, geminin ablation was found to impair the proliferation of normal or immortalized cells, indicating that geminin could potentially serve as a therapeutic target for cancer treatment [16]. Elevated expression of geminin was found in oncogenic activities, which exhibited correlation with invasion of a variety of cancers. Germinin expression was altered in cervical carcinoma and significantly affects cancer prognosis [17]. The present study was conducted to explore the prognostic value of HPV 16/18 genotyping and geminin mRNA quantification in predicting the progressiveness of LSIL.

## Material and methods

### Patientsand tissues

Patients with LSIL, HSIL or cervical cancer who were admitted to Jiangxi Cancer Hospital from December 2016 to November 2019 were recruited for this study.

Women with HSIL, LSIL/CIN1 had been diagnosed by colposcopy-guided biopsy. The inclusion criteria: (1) Within 3 days, no sexual intercourse was conducted, no vaginadouche or administration was performed; (2) Nohistory of CIN diagnosis; (3) Normal liver and kidney function. Exclusion criteria: (1) complication of other morbidities such as diabetes, chronic kidney disease, and heart failure; (2) administrated with immune inhibitor drugs; (3) women in pregnancy or lactation period. In total, we recruited 212 participants, in which 76 are negative for intraepithelial lesion or malignancy, 85 were diagnosed with LSIL, 36 were HSIL, and 15 cervical cancer patients. In LSIL patient group, the mean age was 38.36 ± 7.32, ranging between 27 and 65 years; the average childbearing was 2.14 ± 0.32 times, and delivery 1.39 ± 0.21 times; in HSIL patient group, the mean age was 39.46 ± 6.83, ranging between 26 and 68 years; the average childbearing was 2.64 ± 0.42, and delivery 1.62 ± 0.25.

Cervical tissues were obtained during the excisional treatment and were fixed in 10% neutral buffered formalin and embedded in paraffin following routine procedures. This study was approved by the Ethic Committee of Jiangxi Cancer HospitalHospital, and informed consent were obtained from all participants.

### HPV genotyping

For HPV genotyping, DNA was extracted from the stored SurePath Pap specimens using the DNeasy kit (catalog no. 69506; Qiagen) according to the manufacturer’s instructions. HPV genotyping was conducted using the the TRUPCR® HPV High Risk (E6/E7) Kit, which is an RT-qPCR assay based on oligonucleotide hydrolysis principle that allows higher specificity and sensitivity of E6/E7 region by primer and probes specific for HPV 14 Genotypes (16/18/31/33/35/39/45/51/52/56/58/59/66 and 68) and simultaneous genotyping of 16 and 18.

### Real-time quantitative PCR

Complementary DNA was synthesized from total RNA using a M-MLV reverse transcription kit (Takara Bio, Dalian, China) with the specific primers of GMNN. cDNA samples were used as templates for amplification reactions performed in a PCR Thermal Cycler Dice Real-Time system with the SYBR PrimeScript PCR kit (Takara Bio).

All qPCRs were performed in triplicate at a reaction volume of 25 µL containing 5 µL of cDNA, diluted at a 1:5 ratio, and mixed with Taqman Universal PCR MasterMix. The following protocol was used for all assays: denaturation (95°C for 10 min) and amplification (95°C for 20 s, 60°C for 1 min) repeated for 40 cycles. The housekeeping genes GUSB (beta glucuronidase) and PGK1 (cGMP-dependent protein kinase (1) were selected as reference genes for quality control of the RNA specimens. This combination of reference genes demonstrated a high stability in expression between groups of normal samples versus HSIL samples. The number of cycles required for the signal to cross the threshold (cycle threshold [Ct] value) for target genes was set at 35 cycles and automatically calculated and recorded by the High-Resolution Melt Software v2.0 for each reaction. Ct levels are inversely proportional to the amount of target nucleic acid in the sample (the lower the Ct level, the greater the amount of target nucleic acid in the sample). For the reference genes GUSB and PGK1, a Ct value above 35 cycles indicates poor RNA quality. Expression of GMNN were analyzed with 2−ΔΔCt method. Each sample was quantified in triplicate, and mean values were adopted for further analysis. Expression of GMNN were normalized to endogenous GPDH control.

### Statistical analysis

SPSS 17.0 software was adopted to perform statistical analysis for the data. Normally distributed data were presented as Mean ± SD. Group comparison was analyzed with Student’s *t*-test. Enumeration data was represented with frequency (%) and analyzed with Chi-square nonparametric test. Variables that were significant in univariate analysis were further testified in multivariate logistic regression. Receiver Operating Characteristic Curve (ROC) was employed to verify the diagnostic effectiveness of our logistic prediction model, from which Area Under Curve (AUC) and cutoff values were derived. *P* < 0.05 was considered statistical significant.

## Results

The basic aim of the study was to explore the prognostic value of HPV 16/18 genotyping and geminin mRNA quantification in predicting the progressiveness of LSIL. We recruited 212 participants who were negative for intraepithelial lesion or malignancy (NILM 76), Low-grade squamous intraepithelial lesion (LSIL 85), high-grade squamous intraepithelial lesion (HSIL 36) and Cervical intraepithelial neoplasia grade cervical cancer grade 3, (CIN3 15) patients. Tissues were obtained during excisional treatment. HPV 16/18 genotyping and geminin mRNA qRT-PCR were performed. HPV 16/18 positivity rate and geminin mRNA level were integrated with the clinical parameters into a multivariate logistic model. Area under curve was yielded based on receiver operation curve derived from this multivariate logistic model. Follow-up visits were performed to LSIL patients with progression.

### HPV 16/18 positivity and geminin expression in normal,LSIL, HSIL and cervical cancer tissues

**Figure 1.**
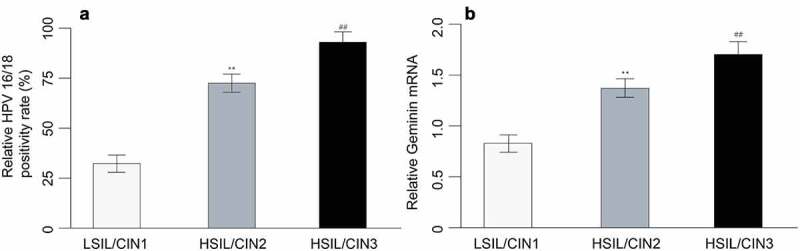
HPV 16/18 positivity rate (a) and geminin mRNA expression in cervix tissues of LSIL/CIN1, HSIL/CIN2 and HSIL/CIN3 participants with a *P* value of less than 0.05

HPV 16/18 mRNA cannot be detected in normal cervical tissues, but was expressed in LSIL, HSIL and cervical cancer tissues (Table 1). The positive rate of HVP16/18 in LSIL, HSIL and cervical cancer tissues were 23.6%, 75%, and 86.7%. The difference of positive rate showed statistical significance. In those HPV 16/18 positive patients, HSIL group exhibited markedly higher HPV 16/18 positivity than LSIL/CIN1 group, and cervical cancer tissues were tested with HPV 16/18 positivity significantly higher than HSIL patients, suggesting that HPV 16/18 load is associated with progression of lesion (Figure 1(a)).

**Table 1.** Clinical parameters of recruited patients

Characteristics		NILM	LSIL	HSIL	Cervical cancer	*p*-value
		*n* = 76	*n* = 85	*n* = 36	*n* = 15	
Age	<30	23	3	0	0	7.35e-08
	≥30	53	82	36	15	
Marital status	Married	58	67	27	11	0.9498
	Single	6	5	2	2	
	Divorced	12	13	7	2	
Parity	Nulligravida	12	8	3	1	0.5296
	Primigravida	21	23	15	5	
	Multigravida	43	54	18	9	
Menstrual Bleeding Pattern	Irregular	33	43	23	2	2.088e-09
	Regular	32	30	7	0	
	Menopause	11	12	6	13	
Postcoital bleeding	No	72	81	34	15	0.8376
	Yes	4	4	2	0	
Current sexual partner	No	8	14	3	7	0.002508
	Yes	68	71	33	8	
Alcohol use	No	69	77	33	14	0.9865
	Yes	7	8	3	1	
Smoking	No	76	84	34	14	0.1981
	Yes	0	2	2	1	
Chronic corticosteroid use	No	72	83	35	15	0.6373
	Yes	4	2	1	0	
History of sexually transmitted diseases	No	64	70	31	12	0.936
	Yes	12	15	5	3	
Pelvic examination	Abnormal	3	2	1	1	0.825
	Normal	73	83	35	14	
Squamocolumnar junction (SCJ visible)	No	15	27	28	15	8.441e-13
	Yes	61	58	8	0	
Lesion size	1–2 (small)	59	55	5	0	0
	3–4 (medium)	4	21	15	1	
	5–8 (large)	0	9	16	14	
HPV 16/18 status	Positive	4	20	27	13	0
	Negative	72	65	9	2	
Geminin mRNA	High	7	26	29	14	0
	low	69	59	7	1	

NILM: negative for intraepithelial lesion or malignancy; SCJ: Squamous columnar junction.

LSIL tissues were detected with low level of geminin, and both HSIL and cervical cancer tissues demonstrated substantially higher geminin mRNA expression (LSIL vs. HSIL, *p* = 0.003; LSIL vs. cervical cancer, *p* = 0.002). HSIL/CIN3 expressed higher geminin than HSIL/CIN2 (Figure 1(b), *p* = 0.001).

### Univariate analysis results of clinical data, expression of HPV16/18 and geminin expression

We further sought to examine the diagnostic value of combining HPV 16/18 and geminin in differentiating LSIL from HSIL. Univariate analysis was performed on the clinical and expressional data of recruited patients (Table 1). No significant difference among NILM, LSIL, HSIL and cervical cancer patients was found in marital status, parity, postcoital bleeding, alcohol use, smoking, chronic corticosteroid use, pelvic examination results and history of sexually transmitted diseases. Significance was found in age, menstrual bleeding pattern, current sexual partner, SCI presence, lesion size, HPV 16/18 status and geminin mRNA expression.

The LSIL, HSIL and cervical cancer participants are mostly elder than 30 years of age. Irregular menstrual bleeding pattern was found in 50% LSIL participants, 63% HSIL patients and 13% cervical cancer patients. 14% NILM, 14% LSIL, 16% HSIL and 86% cervical cancer patients are menopause. Squamocolumnar junction (SCJ) is visible in most of NILM and LSIL participants, but only present in 22% (8/36) of HSIL participants and not even visible in all cervical cancer patients. Overall, HSIL and cervical cancer patients had larger proportion of medium/large size lesions as per TNM classification. A total of 64.7% LSIL patients have small lesion, 24.7% have medium size lesion, and 10.6% have large size lesion. In HSIL group, 13.9% have small lesion, 41.7% have medium size lesion, and 44.4% have large lesion. In addition to the substantial difference of HPV 16/18 positivity mentioned above, high geminin level was found in 30.6% LSIL participants, 80.6% HSIL and 93.3% cervical cancer patients.

### Multivariate logistic regression analysis of clinical data, HPV16/18 and geminin expression

Variables that demonstrated significant difference between the disease groups have been assessed by the multivariate logistic regression model. Results showed that HPV 16/18 positivity status, geminin expression, SCJ presence and lesion size are independent predictor that differentiating LSIL and HSIL, in which HPV 16/18 status and geminin rank as top 2 factors (Table 2). We next calculated the detective effectiveness of this multivariate logistic model by deriving an ROC curve. The AUC was 0.932 for logistic model, with sensitivity of 0.912 and specificity of 0.897, suggesting a remarkable detecting power (Table 3).

**Table 2.** Multivariate logistic regression analysis for prediction of progressive LSIL

Parameter	*B*	SE	Wald	OR	95% CI	*P* value
HPV 16/18 positivity	0.759	0.381	20.559	2.143	1.528–4.291	0.001
Geminin expression	1.406	0.121	13.492	2.082	1.862–3.795	0.002
Menstrual bleeding pattern	0.759	0.381	15.382	1.029	0.483–2.215	0.283
Current sexual partner	0.218	0.048	8.125	1.208	1.024–1.523	0.162
SCJ presence	0.423	0.733	6.124	1.521	1.235–2.103	0.003
Lesion size	0.472	0.641	9.873	1.593	1.202–2.124	0.018

**Table 3.** ROC curve values for logistic model predicting progressive LSIL

Parameter	AUC	*P* value	95% CI	Sensitivity	Specificity
Logistic	0.932	0.000	0.851–0.965	0.912	0.897
HPV 16/18 positivity	0.823	0.001	0.632–0.886	0.728	0.818
Geminin expression	0.882	0.002	0.671–0.942	0.837	0.862
Menstrual bleeding pattern	0.586	0.073	0.467–0.792	0.313	0.452
Current sexual partner	0.478	0.052	0.373–0.643	0.432	0.527
SCJ presence	0.621	0.003	0.531–0.735	0.635	0.576
Lesion size	0.515	0.042	0.412–0.674	0.502	0.482

### Follow-up of LSIL patients revealed correlation between disease progression and expression of HPV 16/18 and geminin

**Figure 2.**
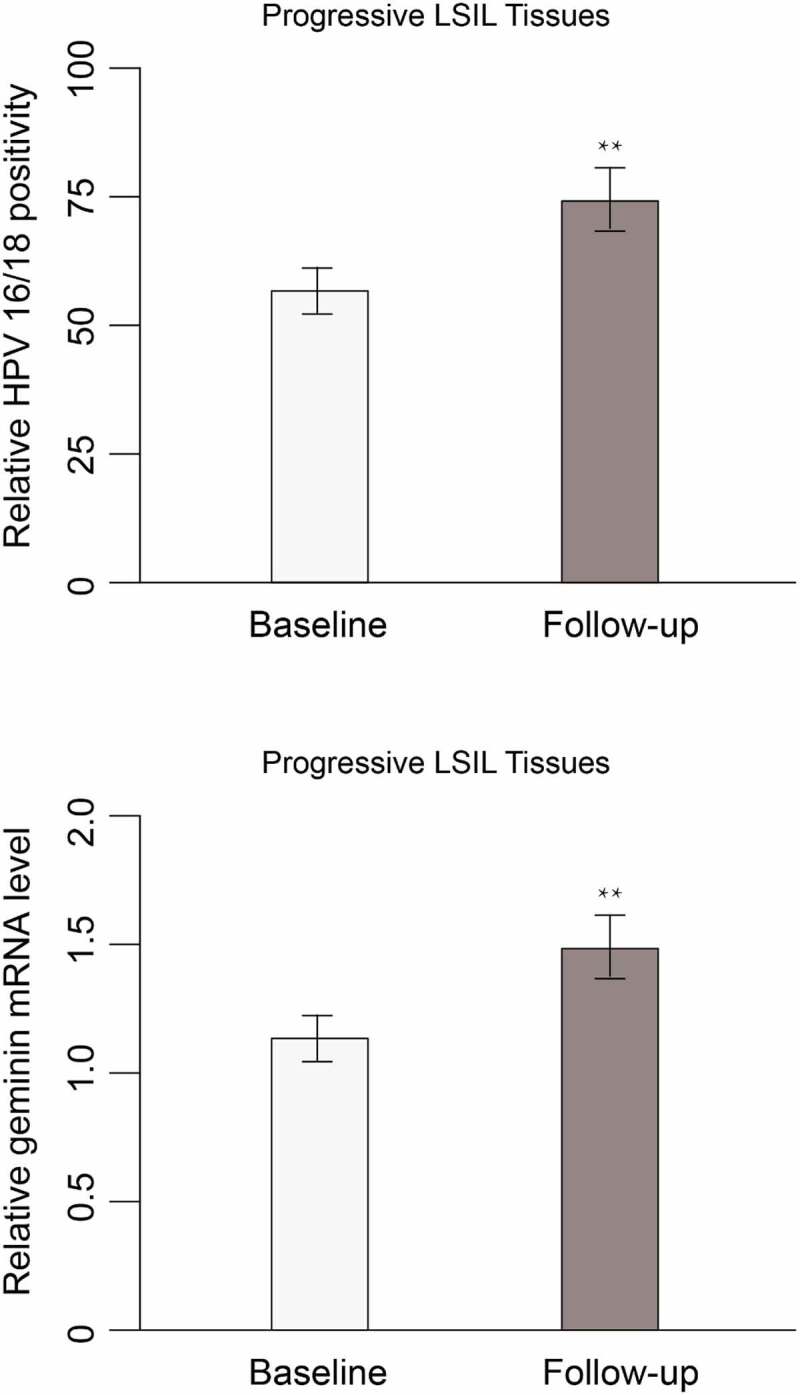
HPV 16/18 positivity rate and geminin mRNA expression in cervix tissues at baseline and follow-up time points of participants with progressive LSIL

Given that HPV 16/18 and geminin hold potential as a prognostic and diagnostic biomarker for differentiating HSIL from LSIL, whether HPV 16/18 and geminin expression change during the progression of LSIL/CIN1 remains to be discovered. Follow-up was done at 6-month intervals for up to 2 years. At each follow-up, women positive for cytology or high-risk HPV (hrHPV) were referred for colposcopy To address this, we followed up 18 women who had LSIL but progressed to HSIL/CIN2 or HSIL/CIN3. Cytology and expression of HPV 16/18 were evaluated. HPV 16/18 positivity and geminin levels were significantly increased as compared with their baseline expression measured (Figure 2). The non-progressive patients have maintained the same or reduced level of baseline HPV 16/18 and geminin expression. LSIL patients who have progress to HSIL have high level of geminin in the tissue (Table 4).

**Table 4.** HPV 16/18 and geminin expression in progressive LSIL

Biomarker	Progressive LSIL, *n* = 18	Non-progressive LSIL, *n* = 67	*p*-value
High HPV 16/18 mRNA	15	5	1.324e-10
Low HPV 16/18 mRNA	3	62	
High geminin level	14	12	4.107e-06
Low geminin level	4	55

## Discussion

Differentiation of LSIL and HSIL can be challenged because the lesion of them are histologically continuous and identifying potential progressive LSIL is even more complicated and requires close follow-up when the progression can be detected. The widely use of LEEP could eradicate the progressive LSIL which further may increase the economic burden to individual, family, and society because of its limited affordability. Therefore, seeking for differentiating biomarker is of great importance for reducing incidence of cervical cancer.

HPVis a heterogeneous group of viruses that are common in human world that comprises of more than 100 types. Among them, 14 types of HPV were found related to cancer, such as cancer of the anus, vulva, vagina, penis and oropharynx [18]. Cervical cancer is the second most common cancer in women in underdeveloped countries. In 2018, over 300,000 women died from cervical cancer and more than 85% of them occurred in third-world countries [19]. Screening and treatment of pre-cancer lesions in women beyond 30 years of age is the most cost-effective way to prevent cervical cancer. Around 70% of cervical cancers and precancerous cervical lesions are caused by HPV 16 and 18. In the present study, we detected higher HPV 16/18 positivity rate in cervical can and HSIL than in LSIL and NILM participants. Among HSIL group, CN3 has significantly higher HPV 16/18 positive rate than CN2. Moreover, in the follow-ups, we found that the progressive LSIL has markedly more proportion of HPV 16/18 positive participants. These results demonstrated HPV 16/18 can be a good prognostic biomarker for differentiating progressive LSIL patients.

Geminin plays an essential role in embryonic development and maintenance of chromosomal integrity. Although it was reported as a protective gene from oncogenesis, multiple lines of evidence demonstrated geminin overexpression in human cancers, suggesting its ambivalent role in cancers. A recent study demonstrated that geminin is aberrantly overexpressed in breast cancer tissues and promotes tumor invasionand metastasis by suppressing FoxO3-mediated transactivation of Dicer [20]. Kim et al. discovered that exogenous and stable expression of GMNN could promote tumorigenesis, gene amplification of GMNN was also shown to be associated with clinical pathophysiological effects in HCC [21]. Montanari et al. demonstrated that geminin overexpression stimulated cell cycle progression and proliferation in both normal and cancer cells and increased the anchorage-independent growth of breast cancer cells [22].Geminin expression is altered in cervical carcinomas also and significantly affects cancer prognosis in addition to other markers such as CDC6 [17]. Zhang et al. employed a network-based strategy to predicate candidate genes implicated in cervical cancer, and GMNN was among the top 20 genes with high weight values in pathogenic network [23]. In the present study, we showed that HSIL/CN3 and HSIL/CN2 have substantially higher mRNA expression level of geminin than LSIL group. In the follow-ups, progressive LSIL patients express higher geminin mRNA in cervix tissues than their baseline levels, while both are also above the average of the whole LSIL group. Such statistical significance suggest that geminin mRNA level could serve as an indicator for prognosis of LSIL.

## Conclusion

We found that HPV 16/18 positivity and geminin level are two independent factors with the most significant OR. The detective effectiveness of this model gauged by the area under curve (AUC) of the receiver operation curve (ROC) yielded an accuracy of 0.932 with sensitivity of 0.912 and specificity of 0.897. Taken together, our study explored the prognostic value of combining HPV 16/18 genotyping and geminin mRNA quantification in differentiation of progressive LSIL, which lends real-world evidence for developing diagnostic tool for LSIL.

## Research highlights

- HPV 16/18 positivity and geminin level are the independent factors with the most significant OR.
- Geminin is implicated in the progression of LSIL.
- In HPV 16/18 positive patients, HSIL group exhibited markedly higher HPV 16/18 positivity than LSIL/CIN1 group

## Limitations

HIV could act as confounding factor in the patients of HPV and can influence the cytological grades.
